# Amusing Scene in an Omnibus

**Published:** 1874-12

**Authors:** 


Amusing Scene in an Omnibus.—A gentleman who is not celebrated for his benevolence, although he is very wealthy, the other evening got into a Fifth Avenue omnibus to go up town. As the stage was full, and he near the door, he reached forward, and touching a man on the shoulder, asked him to pass a one-dollar bill up to the driver for his fare, remarking, "I'm sorry to trouble you, but I have no change." The person to whom this request was made smiled, bowed, and taking a small book from his pocket, wrote something in it, and put the dollar bill in his pocket. All the passengers smiled audibly, and the gentleman demanded his money with one or two expressions such as "fool," "swindler," etc. The person alluded to smiled and bowed again, but did not give up the dollar. The passengers laughed louder. The gentleman grew furious, and pulled the strap, shouting, "Driver, driver! call a policeman." Suddenly, the man with the note-book, his face glowing with pleasure, handed the gentleman a slip of paper, on which was written, "I am deaf and dumb. I thank you for your generosity." The joke was too much for old stagy. He laughed with the rest, and, telling the driver to go ahead, passed up a second dollar, with his own hand, to the driver to be changed, the deaf and dumb man seeming to be as much amused as the other passengers.—*New York Letter.*

